# Supplementary material for: Complex Intervention Programs Integrating Multiple Intervention Strategies Were Not More Effective than Active Control Groups: Evidence from Randomized Controlled Trials
Source: Behav Sci (Basel). 2025 Nov 14;15(11):1554. doi: 10.3390/bs15111554 (PMC12649539; doi:10.3390/bs15111554)
Supplement: Supplementary file 1 [file behavsci-15-01554-s001.zip › behavsci-3808474-supplementary.pdf]

Table S1 Scientific articles topics for active control groups.

Day	Study 1	Study 2
1	emotionally sensitive	sleep trivia
2	compassion fatigue	sleep cycle
3	anxiety	sleep issues
4	accepting depression	biological clock
5	emotional suppression schema	duration vs quality
6	depression	difficulty falling asleep
7	complex post-traumatic stress disorder (CPTSD)	late-night procrastination
8	anxiety	addressing procrastination
9	depression	pre-sleep arousal
10	automated thinking	sleep myths
11	anxiety	how to sleep better
12	the therapeutic effects of melancholic music	bedtime routine
13	how reason and emotion coexist	bedtime routine 2
14	the therapeutic effects of music	advanced techniques

Table S2 Estimated marginal means and 95% confidence intervals for group × time effects for Study 1.

Group	Time	M	SD	95% CI	
				Lower	Upper
Depression					
Intervention	1	62.303	0.847	60.620	63.985
	2	45.662	1.249	43.181	48.143
	3	42.210	1.198	39.831	44.590
Control	1	61.704	0.838	60.039	63.368
	2	43.882	1.236	41.427	46.337
	3	39.304	1.186	36.950	41.658
Cognitive flexibility					
Intervention	1	55.792	1.484	52.845	58.739
	2	57.182	1.451	54.300	60.064
	3	62.643	1.605	59.455	65.830
control	1	57.326	1.468	54.410	60.242
	2	61.985	1.436	59.133	64.837
	3	67.330	1.588	64.176	70.484
Rumination					
Intervention	1	55.792	1.484	52.845	58.739
	2	57.182	1.451	54.300	60.064
	3	62.643	1.605	59.455	65.830
control	1	57.326	1.468	54.410	60.242
	2	61.985	1.436	59.133	64.837
	3	67.330	1.588	64.176	70.484
Perceive stress					
Intervention	1	25.620	0.794	24.043	27.197
	2	21.472	0.798	19.888	23.056
	3	19.850	0.926	18.011	21.689
control	1	25.964	0.786	24.404	27.525
	2	20.476	0.789	18.909	22.044
	3	17.637	0.916	15.817	19.457
Temporal pleasurable experience					
Intervention	1	76.720	1.927	72.893	80.547
	2	79.871	1.911	76.075	83.667
	3	85.294	1.810	81.700	88.888
control	1	80.234	1.907	76.447	84.020
	2	83.025	1.891	79.268	86.781
	3	89.100	1.791	85.543	92.656
Depression stigma					
Intervention	1	34.913	1.494	31.946	37.880
	2	33.620	1.628	30.387	36.853
	3	34.127	1.835	30.484	37.771
control	1	38.942	1.478	36.007	41.878
	2	37.617	1.611	34.418	40.817
	3	34.773	1.816	31.168	38.378

Table S3 Estimated marginal means and 95% confidence intervals for group × time effects for Study 2.

Group	Time	M	SD	95% CI	
				Lower	Upper
Sleep quality problem					
Intervention	1	10.926	0.393	10.147	11.704
	2	9.037	0.368	8.307	9.767
	3	7.537	0.340	6.863	8.212
Control	1	11.518	0.386	10.753	12.282
	2	9.589	0.362	8.872	10.306
	3	8.554	0.334	7.891	9.216
Negative affect					
Intervention	1	22.019	1.766	18.518	25.519
	2	16.944	1.711	13.553	20.336
	3	13.981	1.604	10.803	17.160
control	1	25.339	1.734	21.902	28.777
	2	18.429	1.680	15.098	21.759
	3	17.643	1.575	14.521	20.764
Rumination					
Intervention	1	51.870	1.671	48.558	55.183
	2	47.833	1.623	44.617	51.050
	3	45.389	1.520	42.375	48.403
control	1	55.482	1.641	52.229	58.735
	2	51.643	1.593	48.485	54.801
	3	50.518	1.493	47.559	53.477
Perceive stress					
Intervention	1	51.870	1.671	48.558	55.183
	2	47.833	1.623	44.617	51.050
	3	45.389	1.520	42.375	48.403
control	1	55.482	1.641	52.229	58.735
	2	51.643	1.593	48.485	54.801
	3	50.518	1.493	47.559	53.477
Sleep procrastination					
Intervention	1	35.370	0.836	33.713	37.028
	2	29.981	0.949	28.101	31.862
	3	28.556	1.015	26.544	30.567
control	1	34.911	0.821	33.283	36.538
	2	31.804	0.932	29.957	33.650
	3	30.214	0.996	28.239	32.189
